# Impact on efficacy of target reduction of two FDA-approved ASO drugs by intracellular glucose levels in *in vitro* cell models

**DOI:** 10.1016/j.omtn.2025.102487

**Published:** 2025-02-15

**Authors:** Le Tra Giang Nguyen, Sherouk M. Tawfik, Jing Jin, Andrea Durwin, Xiao-bo Zhong

**Affiliations:** 1 Department of Pharmaceutical Sciences, School of Pharmacy, University of Connecticut, Storrs, CT 06269, USA

**Keywords:** MT: Oligonucleotides: Therapies and Applications, antisense oligonucleotide, ASO, diabetes, glucose, therapeutic efficacy

## Abstract

Antisense oligonucleotides (ASOs) have emerged as a new therapeutic modality for the treatment of both rare and common human diseases. A significant proportion of the patient population that may benefit from ASO therapy may also have common diseases, such as diabetes mellitus. The potential influence of prevalent diseases on the effectiveness of ASO drugs in silencing their target mRNAs remains largely unexplored. The present study utilized *in vitro* cell models to determine the impact on the efficacy of target reduction of two US Food and Drug Administration (FDA)-approved ASO drugs by intracellular glucose levels. Using inotersen and mipomersen as the FDA-approved ASO model drugs, this study demonstrated that a higher intracellular level of glucose resulted in decreased silencing efficacy of target reduction of inotersen and mipomersen in HepG2 cells. Reducing intracellular glucose levels in HepG2 cells, either by knocking down the glucose transporter GLUT2 or by treating with the antidiabetic drug metformin, reversed the decreased silencing efficacy of inotersen and mipomersen. This study brings to light the first indication about the significant impact of intracellular glucose levels on the silencing efficacy of the FDA-approved ASO drugs in an *in vitro* model.

## Graphical abstract

**Figure:**
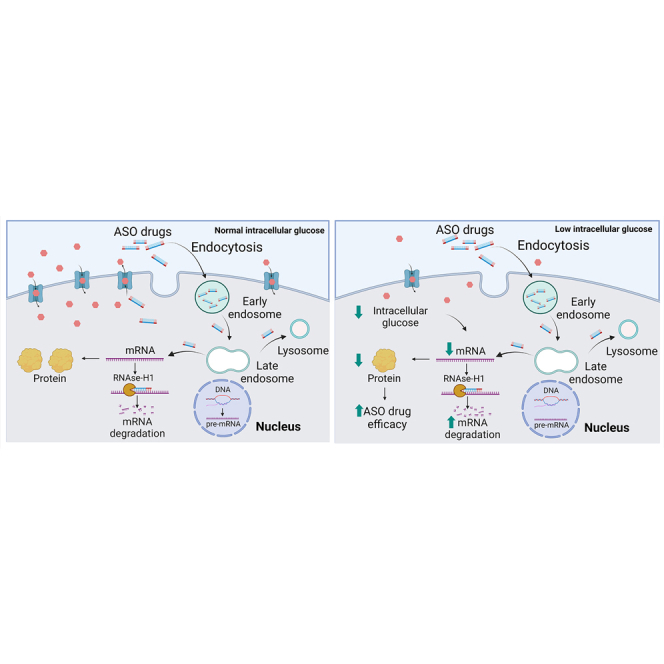

## Teaser

Zhong and colleagues demonstrated that intracellular glucose levels can impact the efficacy of target silencing by the FDA-approved ASO drugs in *in vitro* models, indicating that more studies are needed to investigate the influence of glucose levels on therapeutic uses of ASO drugs for patients with disease conditions, such as diabetes.

## Introduction

An antisense oligonucleotide (ASO) is a small (approximately 18–30 nt) single-stranded RNA-DNA molecule that can complementarily bind to a pre-mRNA or mRNA molecule in a target-specific manner to alter pre-mRNA splicing or mRNA degradation, subsequently altering protein production.^1^ After the discovery of the mechanism, ASOs have shown significant potential to be a new class of modality for bring transformative treatments on human diseases. To date, 14 ASO drugs have been approved by the US Food and Drug Administration (FDA) and/or the European Medicines Agency for the treatment of a variety of human diseases,^2^^,^^3^ particularly for some rare diseases, such as Duchenne muscular dystrophy,^4^ for which small-molecule drugs are not available. It is expected that with more patients benefitting from ASO treatment, some patients may also have common diseases, such as diabetes, hypertension, and hyperlipidemia. The potential influence of pathological states on the efficacy of ASO therapeutics remains largely unexplored. Similar to small-molecule drugs, ASO drugs may exhibit significant interindividual variability in their pharmacokinetic and pharmacodynamic profiles. This variability is likely modulated by multiple factors, including underlying disease conditions.^5^

Diabetes mellitus is a chronic common disease, caused either by insufficient production of insulin by the pancreas or by ineffective use of insulin produced by the body. Hyperglycemia, known as high blood sugar or high blood glucose, is a common symptom of uncontrolled diabetes, leading to severe damage of nerves and blood vessels. Currently, diabetes, cardiovascular diseases, cancer, and chronic respiratory diseases are the four main noncommunicable diseases, causing serious healthcare burden.^6^ This population of patients may benefit from the development of ASO drugs for the treatment of other diseases in the future. However, it remains unclear how diabetes patients with hyperglycemia will respond to ASO drugs differently. This study aims to use *in vitro* cell models to elucidate the impact of intracellular glucose levels on the efficacy and activity of FDA-approved ASO drugs on target reduction.

Two FDA-approved ASO drugs are selected as model drugs in this study, including inotersen and mipomersen. Inotersen, developed by Ionis Pharmaceuticals, was approved by the FDA in 2018 for the treatment of the hereditary transthyretin-mediated amyloidosis (hATTR) in adults.^7^ hATTR is a rare disease caused by multisystem extracellular deposition of amyloid of mutated TTR protein produced by the liver, leading to dysfunction of different organs and tissues, such as the heart, peripheral neuropathy, and eyes.^8^ The principle of drug action (PDA) of inotersen is based on its capability to inhibit the hepatic production of TTR protein via RNase H-mediated mRNA degradation.^9^ Mipomersen, also developed by Ionis Pharmaceuticals, was approved by the FDA in 2013 for the treatment of homozygous familial hypercholesterolemia.^10^ Its PDA also relies on RNase H-mediated mRNA degradation on ApoB-100 and inhibition of ApoB-100 protein translation in the liver.^11^ Reduction of TTR or ApoB-100 at both mRNA and protein levels in the treatment of inotersen or mipomersen was used to access the impact of ASO-mediated target reduction by glucose levels in *in vitro* cell models in the present study.

Two *in vitro* cell models were selected in this study, including HepG2 and HepaRG. HepG2 is a commonly used human hepatoma cell line that has high proliferation rates and retains most hepatic functions, except for low expression of metabolic enzymes.^12^ HepG2 cells have been used as an *in vitro* model to study the reduction of TTR by inotersen.^13^ In contrast to HepG2 cells, which contain only epithelial-like populations, HepaRG is a human bipotent progenitor cell line, which is able to differentiate into two different cell populations, including cholangiocyte-like cells (CLCs) and hepatocyte-like cells (HLCs).^14^ Due to its similarities to primary human hepatocytes (PHHs), HepaRG is considered to be a better compatible alternative to *ex vivo*-cultured PHHs, particularly for drug evaluation and metabolism studies, due to its high expression of metabolic enzymes.^15^ HepG2 and HepaRG cells were selected in this study to evaluate the impact of intracellular glucose levels on the efficacy of ASO drugs of inotersen and mipomersen targeting the liver.

## Results

### Assessment on efficacy of FDA-approved ASO drug-mediated target reduction in different *in vitro* models

**Figure 1:**
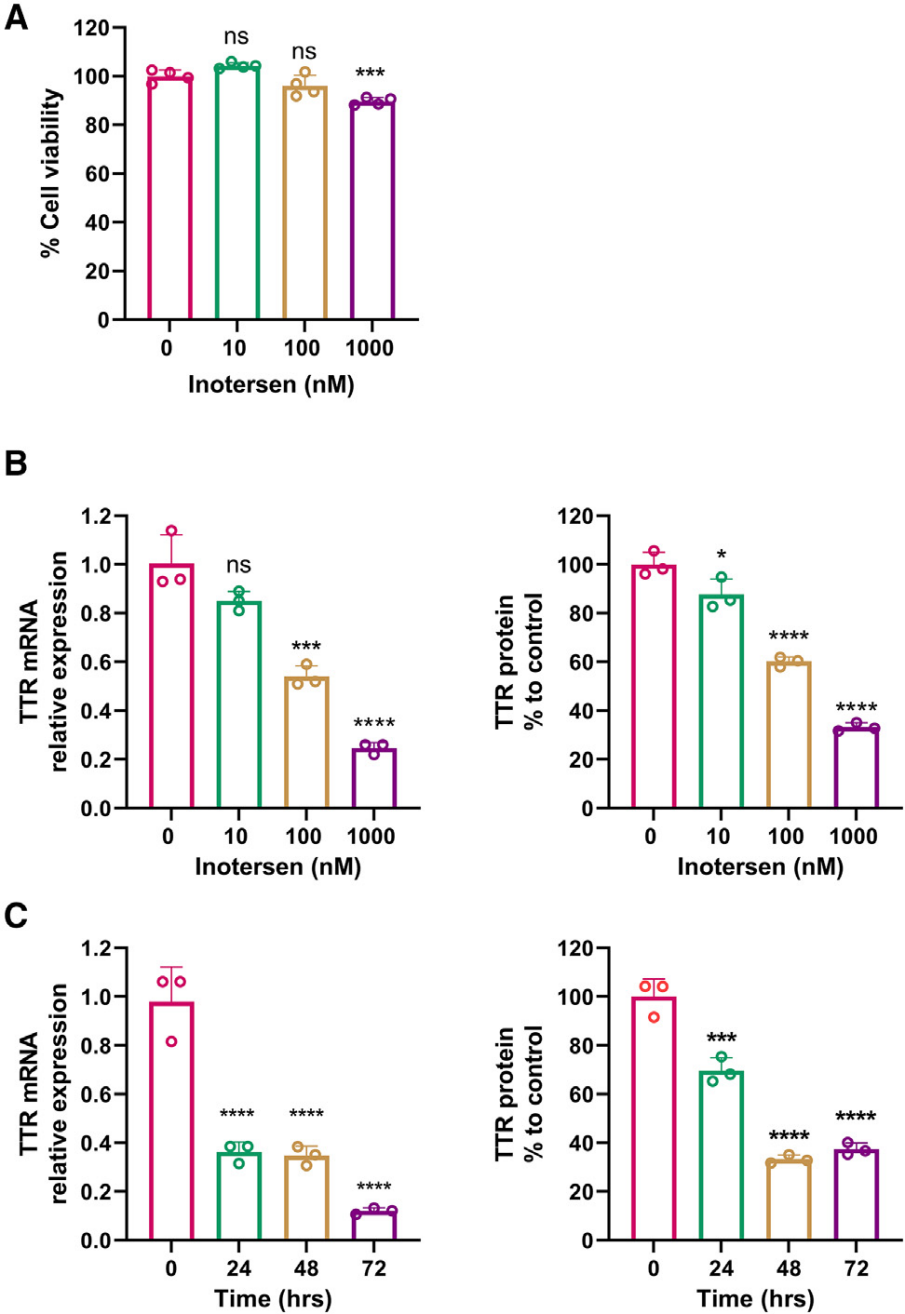
Assessment of the efficacy of inotersen-mediated TTR reduction in HepG2 cells (A) HepG2 cell viability measured by an MTT assay after treatment of 0, 10, 100, and 1,000 nM of inotersen for 48 h. (B) Dose-dependent response on the TTR reduction at mRNA levels (left) and protein levels (right) in treatment with a culture medium containing inotersen at concentrations of 0, 10, 100, and 1,000 nM for 48 h in HepG2 cells. (C) Time-dependent response on the TTR reduction at mRNA levels (left) and protein levels (right) in treatment with a culture medium containing inotersen at 1,000 nM for 0, 24, 48, and 72 h in HepG2 cells. Data are expressed as mean ± SD (*n* = 3). Comparisons were made between each dose and 0 and each time point and 0 with one-way ANOVA test, followed by Tukey post hoc test; ∗*p* < 0.05; ∗∗∗*p* < 0.001; ∗∗∗∗*p* < 0.0001. TTR, transthyretin.

An assessment of the efficacy of FDA-approved drug-mediated target reduction was first performed with an ASO drug, inotersen, in an *in vitro* cell model, HepG2. Initially, a 3-(4,5-dimethylthiazol-2-yl)-2,5-diphenyltetrazolium bromide (MTT) assay was conducted to assess cell viability under the treatment of inotersen. The result showed that the treatment of inotersen did not affect cell health, although a dose of 1,000 nM inotersen that caused a reduction in cell viability to approximate 88% of cells remained viable, which indicated a healthy cell culture environment (Figure 1A). A dose-dependent response on TTR reduction at both mRNA and protein levels to the treatment of inotersen was observed in the HepG2 cell model (Figure 1B). The treatment of inotersen at 10 nM resulted in approximately 20% reduction in TTR mRNA, while reductions of about 50% and 75% were observed at 100 and 1,000 nM, respectively (Figure 1B, left panel). Similar reductions were noted in TTR protein concentrations measured by an enzyme-linked immunosorbent assay (ELISA) in the culture medium released by the HepG2 cells (Figure 1B, right panel). Consequently, all three doses of inotersen (10, 100, and 1,000 nM) were selected for later experiments.

A time course study was performed to identify the optimal time points for assessment of efficacy on TTR reduction by inotersen (Figure 1C). HepG2 cells were treated with inotersen at 1,000 nM for 24, 48, and 72 h. This result showed that a treatment of inotersen for 24 h could result in a significant decrease in TTR mRNA to 40% (Figure 1C, left panel); however, the reduction of TTR protein needed at least 48 h to reach significant reduction to 40% (Figure 1C, right panel). Treatment of inotersen for 48 h was then selected for the following experiments.

**Figure 2:**
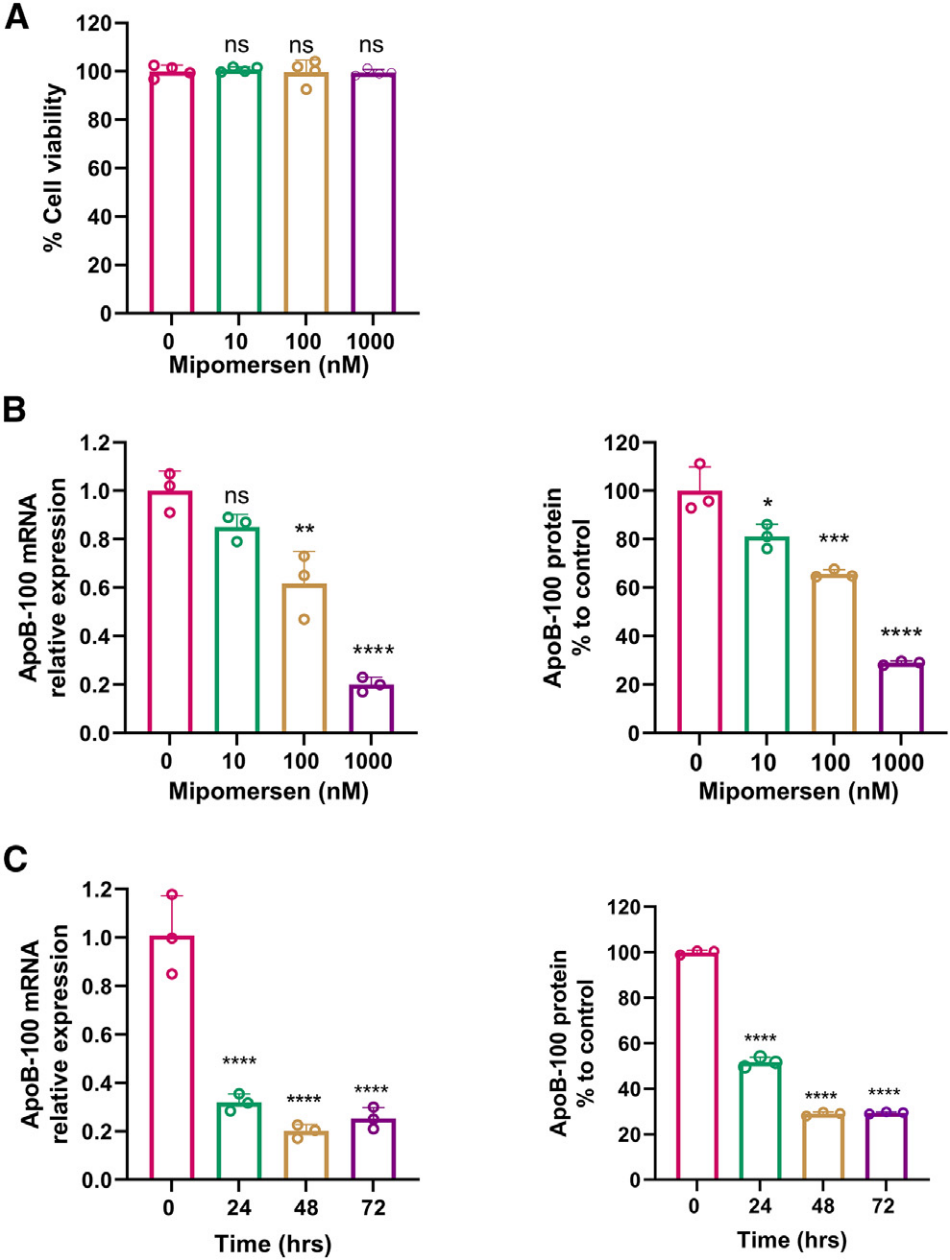
Assessment of the efficacy of mipomersen-mediated ApoB-100 reduction in HepG2 cells (A) HepG2 cell viability measured by the MTT assay after treatment of 0, 10, 100, and 1,000 nM of mipomersen for 48 h. (B) Dose-dependent response on the ApoB-100 reduction at mRNA levels (left) and protein levels (right) in treatment with a culture medium containing mipomersen at concentrations of 0, 10, 100, and 1,000 nM for 48 h in HepG2 cells. (C) Time-dependent response on the ApoB-100 reduction at mRNA levels (left) and protein levels (right) in treatment with a culture medium containing mipomersen at 1,000 nM for 24, 48, and 72 h in HepG2 cells. Data are expressed as mean ± SD (*n* = 3). Comparisons were made between each dose and 0 and each time point and 0 with one-way ANOVA test, followed by Tukey post hoc test; ∗*p* < 0.05; ∗∗*p* < 0.01; ∗∗∗*p* < 0.001; ∗∗∗∗*p* < 0.0001. ApoB-100, apolipoprotein B-100.

The same experimental design was also performed on the second ASO drug, mipomersen, to ensure all observed phenotypes were not specific only to one ASO drug. HepG2 cells also responded to mipomersen treatment in a dose-dependent and time-dependent manner, similar to inotersen (Figure 2). The treatment of mipomersen at 10, 100, and 1,000 nM did not lead to any cytotoxicity for HepG2 cells (Figure 2A). The first bar in Figure 2A was HepG2 cells treated with lipofectamine only as a control, which is the same control presented in the first bar in Figure 1A. Gradual reduction at both mRNA and protein levels of ApoB-100 was observed in HepG2 cells treated with mipomersen at 10, 100, and 1,000 nM (Figure 2B). The treatment of mipomersen at 1,000 mM showed an 80% reduction in mRNA (Figure 2B, left panel) and 75% of protein levels (Figure 2B, right panel). Mipomersen also needed 24 h for a significant reduction at the mRNA level (Figure 2C, left panel), but at least 48 h for protein (Figure 2B, right panel). In conclusion, similar patterns on the dose- and time-dependent responses to the FDA-approved ASO drugs of inotersen and mipomersen on their target mRNA and protein reduction were observed in the *in vitro* HepG2 model.

**Figure 3:**
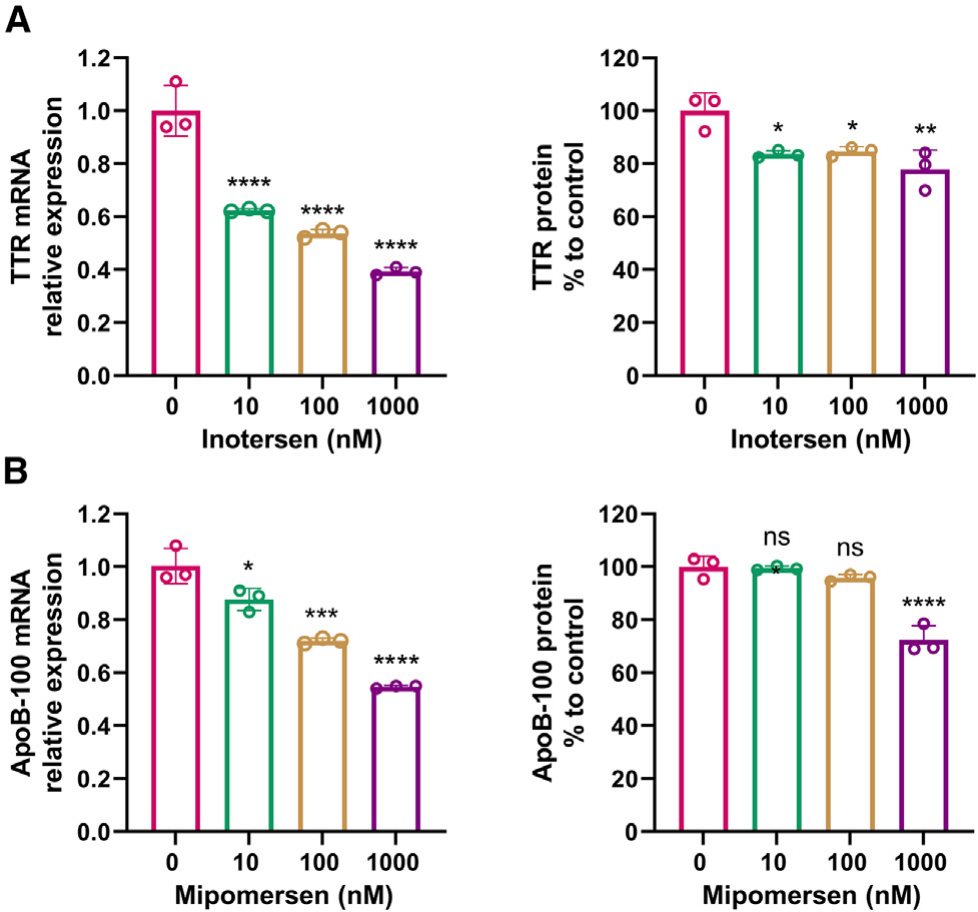
Assessment of the efficacy of ASO drug-mediated target reduction in HepaRG cells (A) Inotersen-mediated TTR reduction in HepaRG cells. (B) Mipomersen-mediated ApoB-100 reduction in HepaRG cells. Data are expressed as mean ± SD (*n* = 3). Comparisons were made with one-way ANOVA test, followed by Tukey post hoc test; ∗*p* < 0.05; ∗∗*p* < 0.01; ∗∗∗*p* < 0.001; ∗∗∗∗*p* < 0.0001.

Another *in vitro* model, HepaRG cells, showed a weaker response on the target reduction with the ASO drugs of inotersen and mipomersen, compared to HepG2 cells (Figure 3). While the reduction on TTR mRNA by inotersen was not significantly different between the two cell lines (Figure 1B, left panel, for HepG2 and Figure 3A, left panel, for HepaRG), TTR protein concentration was reduced by about 20% in HepaRG cells (Figure 3A, right panel), compared to a 70% reduction in HepG2 cells (Figure 1B, right panel) treated with inotersen at 1,000 nM. A similar trend was confirmed with mipomersen. Its efficacy in reducing ApoB-100 mRNA and protein was notably lower in HepaRG (Figure 3B) than in HepG2 cells (Figure 2B). This phenotype could be attributed to the preferential accumulation of ASO drug in non-parenchymal liver cells.^2^ Since HepaRG cells contain both non-parenchymal CLC and parenchymal HLC populations, it might require higher doses of ASO to saturate ASO uptake sites in these non-parenchymal cells before achieving similar significant drug accumulation in HLCs that observed in HepG2. Thus, HepG2 was selected as a proper *in vitro* model for studying the impact on the efficacy of ASO-mediated target reduction by intracellular glucose levels.

### Impact on efficacy of ASO-mediated target reduction by intracellular glucose levels in HepG2 cells

With the aim of understanding the impact of glucose concentrations on the efficacy of ASO drugs, different levels of glucose were chosen for further investigation. The glucose concentrations in the culture media were selected to mimic a low glucose level (2.5 mM), a normal glucose level (5.5 mM), and high glucose levels for prediabetic (10.0 mM) and diabetic (25.0 mM) conditions in the experimental models of diabetes.^16^ First, the MTT assay was performed to evaluate the impact of different glucose levels in the cell culture media on cell viability prior to measuring its impact on intracellular glucose levels. Second, HepG2 cells were incubated with different levels of glucose in cell culture media for 24 h, followed by the treatment of the ASO drugs at 100 nM for an additional 48 h. Efficacy on target reduction was assessed by quantitative real-time polymerase chain reaction for mRNA levels and an ELISA assay for protein concentrations.

**Figure 4:**
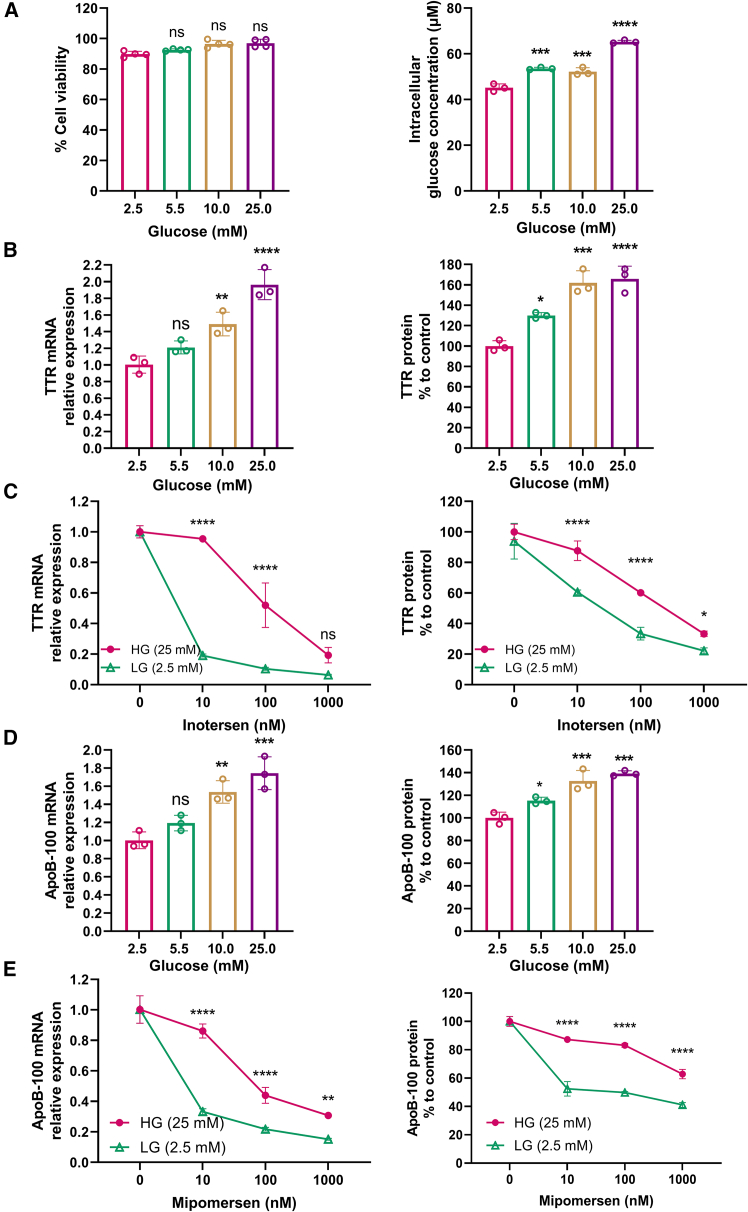
Impact on efficacy of ASO drug-mediated target reduction by glucose concentrations in a culture medium in HepG2 cells (A) HepG2 cell viability measured by the MTT assay (left) and intracellular glucose concentrations (right) after the incubation with a culture medium containing glucose at 2.5, 5.5, 10.0, and 25.0 mM for 24 h. (B) Relative TTR mRNA levels (left) and relative TTR protein concentrations in a culture medium secreted by HepG2 cells (right) after a treatment with a culture medium containing glucose at 2.5, 5.5, 10.0, and 25.0 mM for 24 h, followed by treatment with inotersen at 100 nM for 48 h. (C) TTR mRNA (left) and TTR protein (right) levels after treatment with inotersen (0, 10, 100, and 1,000 nM) for 48 h following treatment with glucose at a high concentration (25 mM) and a low concentration (2.5 mM) for 24 h. (D and E) Same treatment as (B) and (C) for the reduction of ApoB-100 by mipomersen. Data are expressed as mean ± SD (*n* = 3). Comparisons were made with one-way ANOVA test, followed by Tukey post hoc test; ∗*p* < 0.05; ∗∗*p* < 0.01; ∗∗∗*p* < 0.001; ∗∗∗∗*p* < 0.0001.

The result from the MTT assay revealed that different glucose levels on the cell culture media did not have any influence on cell viability and cytotoxicity (Figure 4A, left panel). In addition, the intracellular glucose changed according to the glucose levels on the cell culture media (Figure 4A, right panel). Efficacy on TTR reduction by inotersen at both mRNA (Figure 4B, left panel) and protein levels (Figure 4B, right panel) is correlated to the glucose concentrations in the culture media. Specifically, HepG2 cells incubated with a normal glucose concentration (5.5 mM) experienced no significant difference in TTR reduction compared to those with a low glucose concentration (2.5 mM) at both mRNA and protein levels. Meanwhile, HepG2 cells incubated with high glucose concentrations at 10.0 and 25.0 mM showed about 1.4- and 1.8-fold less effectiveness in TTR mRNA reduction by inotersen, respectively, compared to the low glucose concentration (2.5 mM) (Figure 4B, left panel). Additionally, HepG2 cells treated with glucose at 5.5, 10.0, and 25.0 mM had significantly lower efficacy on TTR protein reduction by inotersen compared to the HepG2 cells treated with glucose at 2.5 mM (Figure 4B, right panel). Furthermore, the dose-response curve of TTR reduction in the treatment with inotersen at 10, 100, and 1,000 nM was shifted significantly in the presence of glucose in the medium at 25.0 mM as compared to 2.5 mM at both mRNA (Figure 4C, left panel) and protein levels (Figure 4C, right panel). These results support the conclusion that higher glucose concentrations in culture media reduce the efficacy of ASO-mediated target reduction.

The conclusion above was also supported by observation from the treatment of mipomersen in HepG2 cells (Figures 4D and 4E). Compared to the low glucose level (2.5 mM) in the culture medium, HepG2 cells incubated with a normal glucose level (5.5 mM) did not show significant changes, while incubation with higher glucose concentrations at 10.0 and 25.0 mM resulted in 1.5- and 1.7-fold decreases in ApoB-100 reduction at the mRNA level by mipomersen (Figure 4D, left panel). At the ApoB-100 protein level, HepG2 cells incubated with increasing glucose levels from 5.5 to 25.0 mM in the culture media significantly diminished the efficacy of mipomersen (Figure 4D, right panel). In addition, across three doses of mipomersen, the high glucose level at 25.0 mM notably attenuated its effectiveness in silencing ApoB-100 at both mRNA (Figure 4E, left panel) and protein levels (Figure 4E, right panel). In conclusion, these results indicate that glucose concentrations in the culture media significantly influence the efficacy of ASO-mediated target reduction for both inotersen and mipomersen.

### Impact on efficacy of ASO drug-mediated target reduction by knocking down glucose transporter 2 protein with siRNAs in HepG2 cells

**Figure 5:**
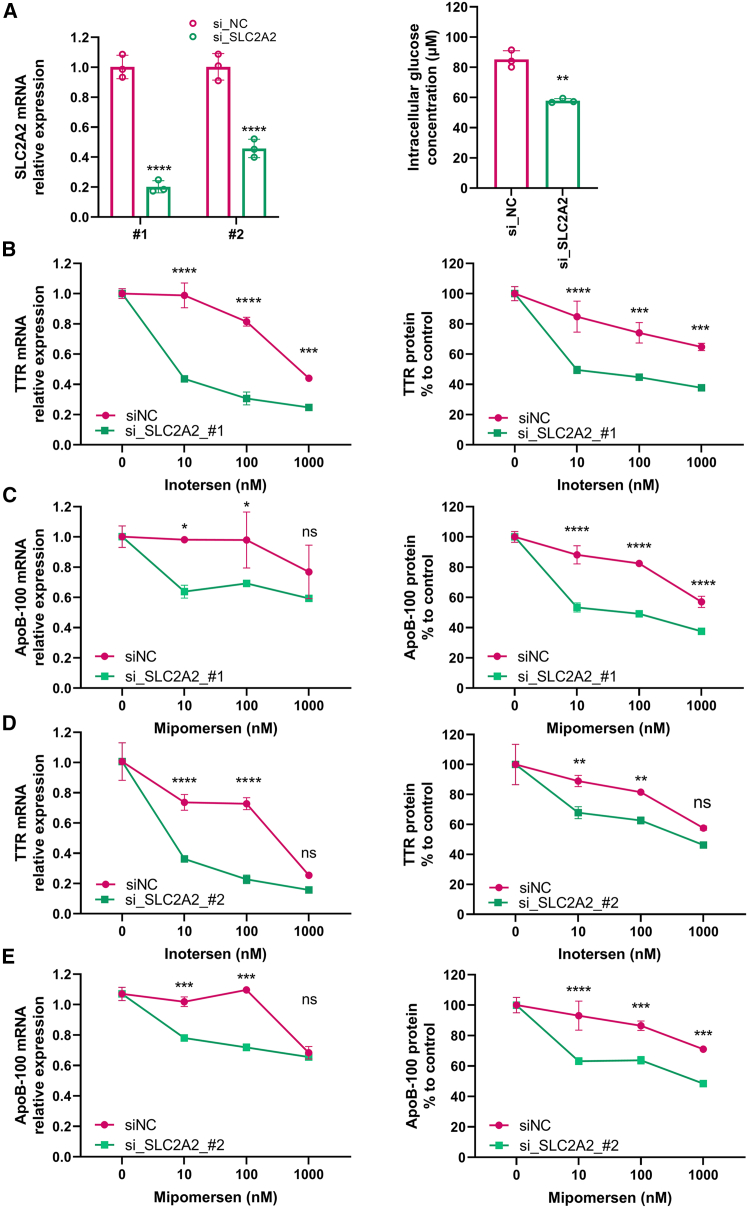
Impact on the efficacy of ASO drug-mediated target reduction by knockdown of GLUT2 protein with siRNAs in HepG2 cells (A) Knockdown efficiency of GLUT2 mRNA using two siRNAs (left) and intracellular glucose concentrations after GLUT2 suppression (right) in HepG2 cells. (B) Reduction of TTR mRNA (left) and protein (right) by inotersen in a dose-dependent manner in si_SLC2A2_#1 in comparison with siNC_#1 in HepG2 cells. (C) Reduction of ApoB-100 mRNA (left) and protein (right) by mipomersen in a dose-dependent manner in si_SLC2A2_#1 in comparison with siNC_#1 in HepG2 cells. (D and E) Same treatment as (B) and (C) using si_SLC2A2_#2 and siNC_#2. Data are expressed as mean ± SD (*n* = 3). Comparisons between si_SLC2A2 and siNC were made with one-way ANOVA test, followed by Tukey post hoc test; ∗*p* < 0.05; ∗∗*p* < 0.01; ∗∗∗*p* < 0.001; ∗∗∗∗*p* < 0.0001.

Glucose transporter 2 (GLUT2) protein, encoded by the solute carrier family 2 member 2 (*SLC2A2*) gene, is a facilitative glucose transporter in the liver, pancreas, intestine, kidney, and brain, which serves as a glucose sensor to maintain glucose homeostasis.^17^ In the liver, GLUT2 is the major transporter that regulates both uptake and efflux of glucose in response to a fed or fasted state.^18^^,^^19^ Consequently, GLUT2 was chosen for further exploration of the impact on the therapeutic efficacy of ASO drugs by glucose levels. To determine whether GLUT2 is important for ASO drug activity, GLUT2 protein levels were transiently knocked down by treating HepG2 cells with two different small interfering RNAs (siRNAs) designed to target GLUT2 mRNA. The results showed that GLUT2 mRNA (Figure 5A) was significantly reduced to approximate 80% and 60% after treatment with two siRNAs target GLUT2, si_SLC2A2_#1 and si_SLC2A2_#2, respectively, compared to their proper scramble siRNA controls (si_NC_#1 and si_NC_#2) for 48 h (Figure 5A, left panel).

To investigate the impact of GLUT2 on intracellular glucose levels, HepG2 cells were lysed and their intracellular glucose contents measured after 48 h treatment with siRNAs targeting GLUT2 mRNA. The result showed that knocking down the GLUT2 transporter significantly reduced intracellular glucose concentrations compared to the control groups (Figure 5A, right panel).

To explore the impact of GLUT2 on the efficacy of ASO drugs, HepG2 cells were first treated for 48 h with si_SLC2A2_#1 and si_SLC2A2_#2. The treated cells were further incubated with ASO drugs at different concentrations for an additional 48 h. The data showed that knocking down GLUT2 resulted in a significantly better silencing efficiency on TTR mRNA in both si_SLC2A2_#1 and si_SLC2A2_#2 (Figure 5B, left panel and Figure 5D, left panel) and protein levels (Figure 5B, right panel and Figure 5D, right panel) at all tested concentrations of inotersen, with the more significant effect observed at lower doses of inotersen such as 10 or 100 nM, compared to the high dose of 1,000 nM. It is also notable that the improvement of inotersen activity was more evident at the mRNA level (Figure 5B, left panel and Figure 5D, left panel), when compared to the protein level (Figure 5B, right panel and Figure 5D, right panel). A similar trend was also observed in mipomersen, as knocking down GLUT2 also enhanced ApoB-100 silencing at both mRNA (Figure 5C, left panel and Figure 5E, left panel) and protein levels (Figure 5C, right panel). These data helped to further confirm the influence of glucose on ASO-mediated target reduction. Also, the enhancement of ASO activity was more significant in the case of si_SLC2A2_#1 compared to si_SLC2A2_#2. It could be because si_SLC2A2_#1 has a better effect when knocking down the GLUT2 transporter.

### Impact on efficacy of ASO-mediated target reduction by an antidiabetic drug, metformin, in HepG2 cells

Since glucose could have some impact on the activity of ASO drugs, there was no evidence on whether medications altering cellular glucose levels can influence the efficacy of ASO drugs. Thus, metformin, the first-line anti-diabetic drug, was chosen for further study. Metformin works by targeting hepatic mitochondria and inhibiting mitochondrial respiratory chain complex I, leading to decrease in ATP synthesis and increase in AMP to ATP ratio in hepatocytes. The mild increase in intracellular AMP levels inhibits AMP-regulated enzymes involved in gluconeogenesis, further decreasing gluconeogenesis.^20^

**Figure 6:**
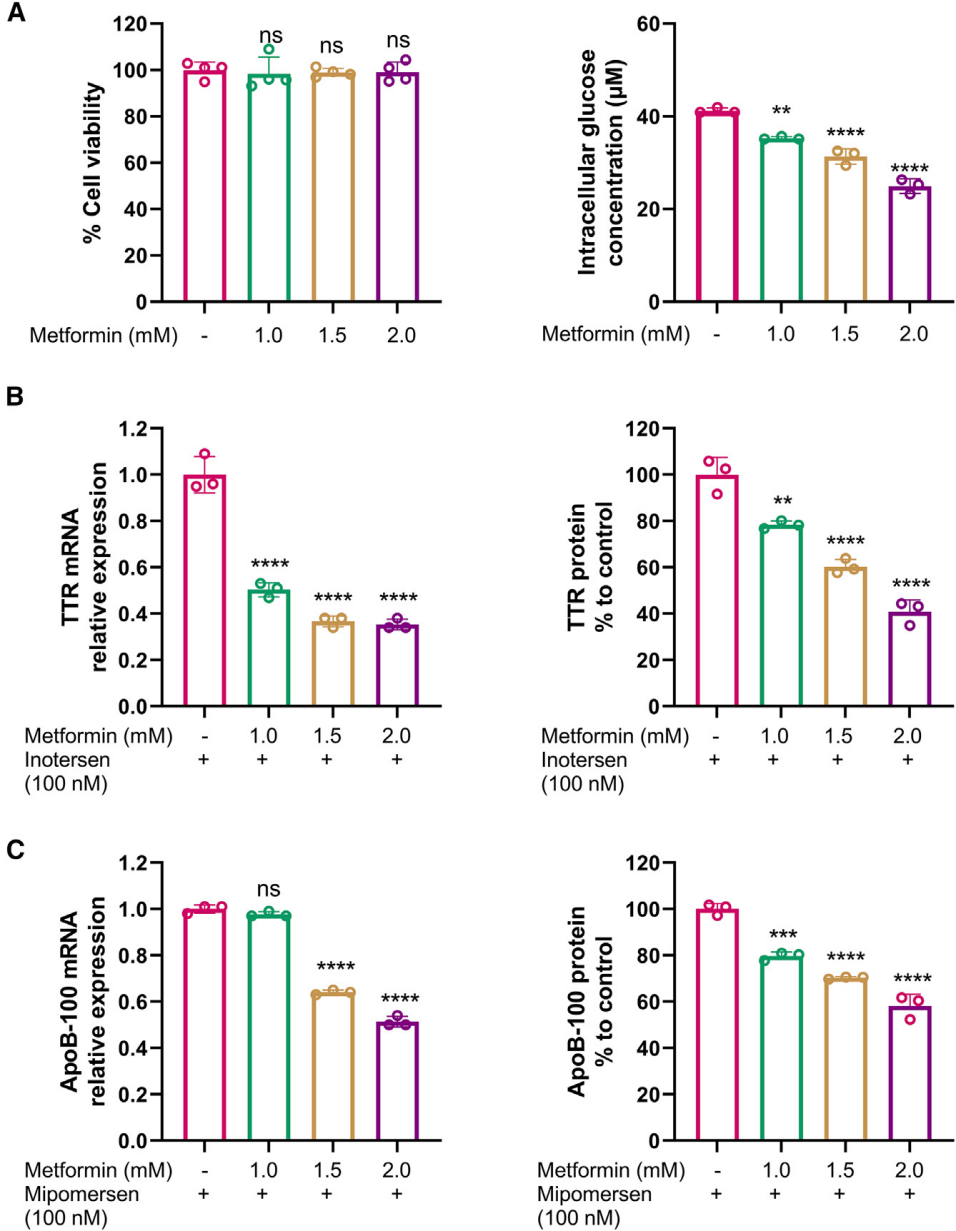
Impact on the efficacy of ASO drug-mediated target reduction by metformin in HepG2 cells (A) HepG2 cell viability measured by the MTT assay (left) and intracellular glucose concentrations (right) after treatment with 1.0, 1.5, and 2.0 mM of metformin for 24 h. (B) Reduction of TTR mRNA (left) and protein (right) by inotersen (100 nM) in the presence of metformin at 1.0, 1.5, and 2.0 mM. (C) Reduction of ApoB-100 mRNA (left) and protein (right) by mipomersen (100 nM) in the presence of metformin at 1.0, 1.5, and 2.0 mM. Data are expressed as mean ± SD (*n* = 3). Comparisons between metformin at different doses and no metformin (0 mM) were made with one-way ANOVA test, followed by Tukey post hoc test; ∗*p* < 0.05; ∗∗*p* < 0.01; ∗∗∗*p* < 0.001; ∗∗∗∗*p* < 0.0001.

Metformin at concentrations of 1.0, 1.5, and 2.0 mM were chosen for investigation based on evidence from previous studies on HepG2,^21^^,^^22^ and these doses of metformin also did not affect cell viability, when compared to the control assessed using the MTT assay (Figure 6A, left panel). To confirm the impact of metformin on intracellular glucose levels, intracellular glucose levels of HepG2 were assessed after the treatment of different doses of metformin. The results showed that increasing doses of metformin significantly reduced intracellular glucose levels (Figure 6A, right panel).

HepG2 cells were treated with metformin at concentrations of 1.0, 1.5, and 2.0 mM for 24 h, followed by incubation with an ASO drug for 48 h. Treatment with metformin at 1.0, 1.5, and 2.0 mM resulted in an approximately 2-fold improvement in the efficacy of inotersen in the reduction in TTR mRNA (Figure 6B, left panel) and protein levels (Figure 6B, right panel), with a more notable effect observed at the mRNA level. In contrast, the reduction in ApoB production at both mRNA (Figure 6C, left panel) and protein (Figure 6C, right panel) levels by mipomersen was only enhanced when HepG2 cells were pretreated with metformin at a concentration of more than 1.5 mM. Therefore, metformin treatment appears to enhance the efficacy of ASO drugs and may be considered to be a potential enhancer in ASO therapy for diabetes patients.

## Discussion

Our study presents a first-step effort to evaluate the impact of glucose levels on efficacy of the FDA-approved ASO drugs of inotersen and mipomersen using two *in vitro* cell models, HepG2 and HepaRG cells, to mimic a diabetic condition. Experimental models have been widely used to study diabetes mellitus and its complications with both advantages and limitations,^23^ including *in vitro*,^24^ *in vivo*,^25^ and microphysiological systems (MPSs), such as organoid and organ-on-chip.^26^ Glucose concentrations in a culture medium can be used to mimic the disease status of diabetes mellitus in an *in vitro* model, with 2.5 mM as low glucose, 5.5 mM as normal glucose, 10.0 mM as pre-diabetic levels, and more than 25.0 mM as a diabetic condition.^27^ The *in vitro* cell models used in this study are helpful for exploring various aspects of diabetes due to their stability and reproducibility.

**Figure 7:**
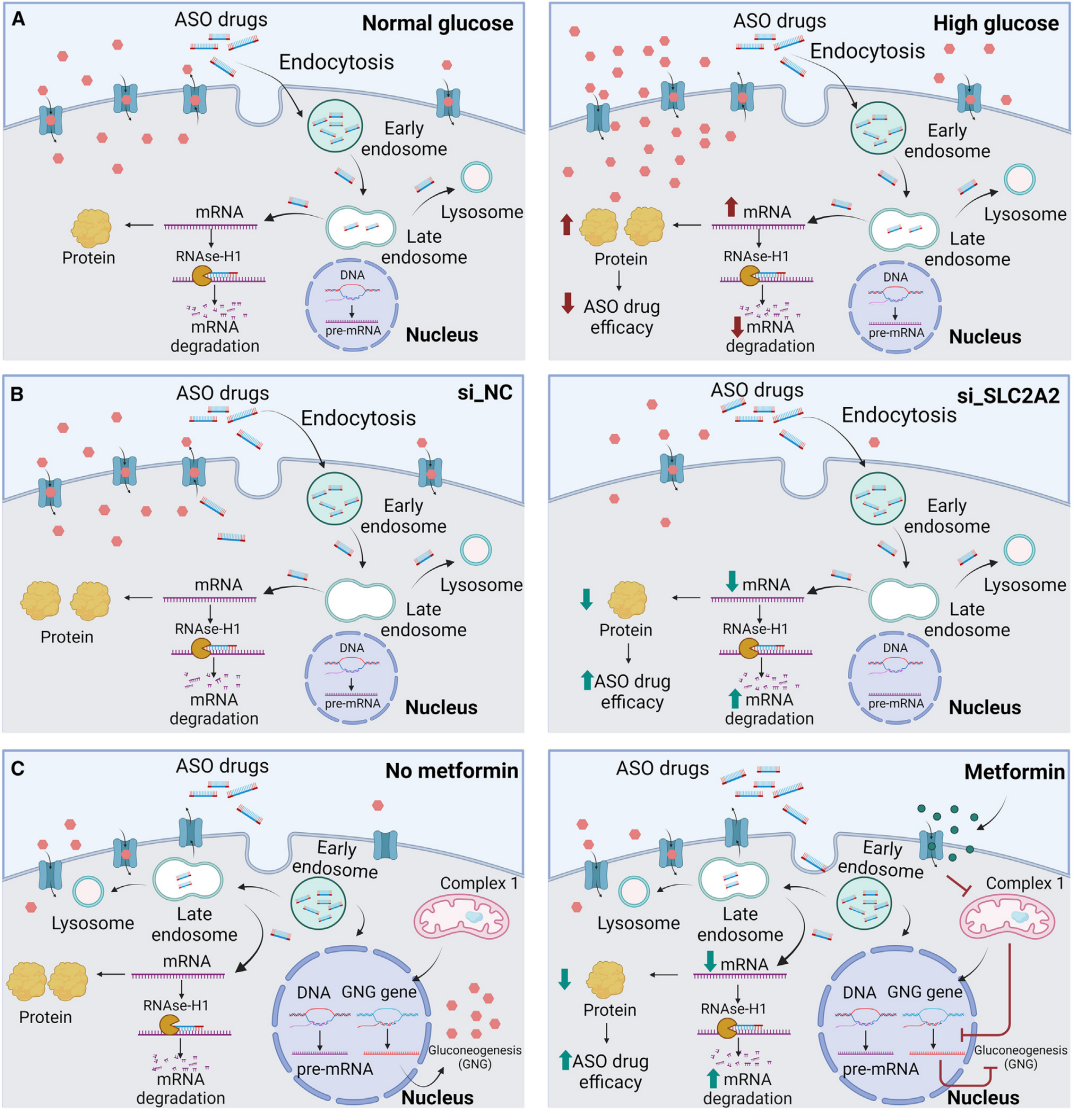
Summary of the impact of glucose levels on ASO-mediated target reduction in *in vitro* cell models (A) A higher glucose level in a cell culture medium reduces efficacy of ASO drugs. (B) Knocking down GLUT2 enhances target silencing by ASO drugs. (C) Metformin treatment improves efficacy of ASO drugs.

As illustrated in Figure 7A, the data presented in this study demonstrate an impact on the silencing efficacy of the FDA-approved ASO drugs by intracellular glucose levels. Under normal glucose conditions (Figure 7A, left panel), after uptake by endocytosis, ASO drugs undergo a series of intracellular trafficking events among membrane compartments via early endosomes to late endosomes to lysosomes. Only a small proportion of the administrated ASO drugs is eventually released from the membrane compartments and available to target their complementary mRNA strand. After binding to their mRNAs forming a DNA-RNA complex, the ASO drugs initiate RNase-H1-mediated degradation of targeting mRNAs to reduce the production of proteins, which are further released from the HepG2 cells into the culture medium, detectable by an ELISA assay. Under high glucose conditions (Figure 7A, right panel), more glucose molecules in the culture medium enter the HepG2 cells by the glucose transporter GLUT2, which results in an increase in cellular levels of the targeted mRNAs and extracellular levels of the targeted proteins, indicating a decrease in the silencing efficacy of the ASO drugs.

The impact on the silencing efficacy of the FDA-approved ASO drugs by intracellular glucose levels is further confirmed by knocking down the glucose transporter GLUT2 in the HepG2 cells (Figure 7B). In a negative siRNA control (si_NC), HepG2 cells express a normal level of GLUT2 to uptake glucose from the culture medium into HepG2 cells (Figure 7B, left panel). In the condition of siRNA knockdown of GLUT2 (si_GLUT2) (Figure 7B, right panel), fewer GLUT2 proteins are available on the HepG2 cell surface, resulting in a lower intracellular glucose level. The decreased ASO-targeting mRNA and protein levels in the knocking down condition indicate an increase silencing efficacy of the ASO drugs, further demonstrating the impact of intracellular glucose levels.

The treatment with an antidiabetic drug, metformin, provides additional evidence to support the findings on the impact of intracellular glucose levels on the silencing efficacy of the FDA-approved ASO drugs (Figure 7C). Without metformin (Figure 7C, left panel), the intracellular glucose levels of HepG2 cells are due to both the uptake of glucose from the medium and the amount of glucose produced by the cells via gluconeogenesis. With the treatment of metformin (Figure 7C, right panel), the intracellular glucose level is reduced by the inhibition of gluconeogenesis by metformin, resulting in an increase in the silencing efficacy of the ASO drugs on the production of both targeted mRNAs and proteins.

In conclusion, this study provides novel insights into the significant influence of intracellular glucose concentrations on the silencing efficacy of FDA-approved ASO drugs in an *in vitro* model. However, it is critical to acknowledge the limitation of this study. The *in vitro* cell models could not fully represent diabetes conditions because they lack systemic interactions between liver, kidney, and vascular systems and simplify the microenvironment with the absence of hormone regulations. Therefore, the present study is only the first step in assessment of the impact of glucose levels on the efficacy of ASO drugs. Further studies will be conducted to better understand the impact of diabetes on the activity of ASO drugs using better experimental models, such as *in vitro* MPSs with connection to multiple organs, such as liver^28^ and liver-kidney organ chips^29^ and *in vivo* animal models.

The underlying mechanisms by which glucose alters the silencing efficacy of the ASO drugs need to be investigated further, which may focus on the impact of glucose on ASO uptake, intracellular trafficking, endosomal release, and target mRNA binding. This knowledge is essential for developing more precision therapeutical approaches targeting various human diseases with ASO drugs. Further research is needed to elucidate these relationships and to develop strategies to enhance ASO efficacy in different metabolic contexts.

## Materials and methods

### Synthesis of inotersen and mipomersen

The same formulations as the FDA-approved inotersen and mipomersen were synthesized by ChemGenes Corporation (Wilmington, MA). The lyophilized ASOs were reconstituted in purified water and stored at −20°C.

### Cell culture of HepG2 and HepaRG cells

HepG2 cells were purchased from the American Type Culture Collection (ATCC, HB-8065, Manassas, VA) and were cultured in a DMEM medium (GIBCO, Grand Island, NY) with an addition of 10% fetal bovine serum (GIBCO, Grand Island, NY). HepaRG cells were provided by Biopredic International (HPR101, Rennes, France) and were cultured following the provider’s protocol. Specifically, HepaRG cells were initially cultured in a HepaRG growth medium including Williams’ E medium (Thermo Fisher Scientific, Carlsbad, CA), GlutaMAX (Thermo Fisher Scientific), and growth additives (ADD710, Biopredic International) for 14 days until the cells became fully confluent. The cells were then kept in a HepaRG differentiation medium with differentiate additives (ADD720, Biopredic International) for 2 more weeks until cells were fully differentiated. Both HepG2 and HepaRG cells were incubated at 37°C and 5% CO_2_, and the medium was renewed every 3 days.

### MTT assay

HepG2 cells were seeded into a 96-well plate for treatment. To measure cell viability, cell culture medium was replaced with 100 μL/well of 1 mg/mL MTT solution (Thermo Fisher Scientific) in a DMEM medium. Cells were then incubated for 1 h at 37°C. MTT, a yellow tetrazolium dye, is reduced by the enzyme mitochondrial dehydrogenase, which is present in the mitochondria of viable cells, into purple formazan. Consequently, the intensity of the color, resulting from the formation of formazan crystals, is proportional to the number of viable cells. Afterward, the MTT solution was removed from the wells, and the formazan crystals were dissolved in 100 μL DMSO per well. Absorbance was measured at 570 nm, and this formula was used to calculate cell viability:

(Equation 1) $$\%\;\text{Viability}=\frac{\text{Absorbance}\;\text{of}\;\text{treated}\;\text{cell}\;–\;\text{absorbance}\;\text{of}\;\text{blank}}{\text{Absorbance}\;\text{of}\;\text{untreated}\;\text{control}\;–\;\text{absorbance}\;\text{of}\;\text{blank}}\times 100$$

### Treatment of cells with the FDA-approved ASO drugs

HepG2 or differentiate HepaRG cells were treated with inotersen or mipomersen at different doses (0, 10, 100, and 1,000 nM) and harvested at different time points (0, 24, 48, and 72 h). After treatment with the ASO drugs, mRNA levels of TTR and ApoB-100 were quantified by quantitative real-time PCR, while protein concentrations secreted by the cells into the culture medium were quantified by ELISA.

### Treatment of the cells with glucose

Glucose (GIBCO) was added to a DMEM medium supplemented with 10% fetal bovine serum to achieve different concentrations with a low level (2.5 mM), a normal level (5.5 mM), and high levels (10.0 and 25.0 mM). HepG2 cells were incubated with a culture medium containing different glucose levels for 24 h, followed by treatment with the ASO drugs for an additional 48 h.

### siRNA knockdown of GLUT2 in HepG2 cells

For siRNA transfection, HepG2 cells were transfected with either si_SLC2A2_#1 or si_SLC2A2_#2 targeting GLUT2 mRNA (s12929 and s12930, Thermo Fisher Scientific) or si_NC (#4404021, Invitrogen, Tewksbury, MA) as a negative control using the Lipofectamine RNAiMAX Transfection Reagent (Thermo Fisher Scientific) following the manufacturer’s protocol. Total RNAs harvested at 48 h after siRNA transfection were used for analysis of knockdown efficiency.

### Treatment of HepG2 cells with metformin

Metformin powder (no. D150959, Sigma, St. Louis, MO) was diluted in purified water to obtain metformin solutions of 1.0, 1.5, and 2.0 mM. Then, HepG2 cells were treated with different concentrations of metformin for 24 h before an incubation with the ASO drugs (100 nM) for 48 h.

### RNA extraction and reverse transcription

Extraction of total RNAs from each cell sample was conducted using TRIzol (no. 15596018, Invitrogen), according to the manufacturer’s instruction. The extracted total RNA was subjected to reverse transcription by the iScript cDNA Synthesis Kit (no. 1708891, Bio-Rad, Carlsbad, CA), following the manufacturer’s protocol.

### Quantitative real-time qPCR

The quantitative real-time qPCR reactions were conducted with an iTaq Universal SYBR Green Supermix (no. 1725121, Bio-Rad) based on the manufacturer’s instruction. β-Aactin was used as a housekeeping gene for quantitative real-time qPCR normalization. The following primer pairs were used: ApoB-100, forward 5′-TCCAGAGAGAGGACAGAGCC-3′ and reverse 5′-TCATGGTAGCCTCAGTCTGC-3’; TTR, forward 5′-AGCCCCTACTCCTATTCCACC-3′ and reverse 5′-ACATGAAATCCCATCCCTCGTC-3′; and GLUT2, forward 5′-ACCACGTCCTGCTGCTTTAG-3′ and reverse 5′-AGGTCCACAGAAGTCCGCAA-3′.

### Quantification of TTR and ApoB-100 proteins by ELISA

An ApoB-100 ELISA kit (EH35RB, Thermo Fisher Scientific) and TTR ELISA kit (ab108895, Abcam, Waltham, MA) were utilized for the quantification of protein concentrations, following the manufacturer’s instructions, with cell culture supernatants being diluted at an appropriate ratio. Absorbance was measured at 450 nm, and protein concentrations were calculated from a reference protein standard curve.

### Intracellular glucose measurement

HepG2 cells with different treatment conditions were washed with PBS buffer before being trypsinized and centrifuged at 1,200 rpm for 5 min. After centrifugation, the cell pellet was washed with PBS twice and resuspended in 1× reaction buffer. The cell pellet was then used to assess the intracellular glucose by an Amplex Red Glucose Assay Kit (Thermo Fisher Scientific) following the manufacturer’s protocol. Absorbance was measured at 560 nm, and glucose concentrations were calculated from a reference standard curve.

### Data analysis

The data are presented as means ± standard deviations (SDs). Statistical analyses for the data were performed using GraphPad Prism (La Jolla, CA). Statistically significant differences in gene expression between two groups were assessed using a one-way ANOVA test. Significant *p* values are indicated with asterisks as follows: ∗*p* < 0.05; ∗∗*p* < 0.01; ∗∗∗*p* < 0.001; ∗∗∗∗*p* < 0.0001.

## Data availability

All raw data and processed data are stored on the OneDrive of the Zhong laboratory at the University of Connecticut. Following the NIGMS Data Management and Sharing Plan policy, all raw data and processed data have been uploaded to Mendeley Data at https://data.mendeley.com/datasets/srn5hc25bt/1.

## Acknowledgments

This study was supported by the 10.13039/100000057National Institute of General Medical Sciences (5R35GM140862 to X.Z).

## Author contributions

L.T.G.N., S.M.T., J.J., and X.Z. design the concept. L.T.G.N., S.M.T., J.J., and A.D. performed the experiments. L.T.G.N., S.M.T., and A.D. analyzed the experimental data. L.T.G.N. processed the data submission. L.T.G.N. and X.Z. wrote the article, with contributions from all authors. The authors have read and approved the final manuscript.

## Declaration of interests

The authors declare no competing interests.
